# Magnetic Resonance Coronary Angiography: Where Are We Today?

**DOI:** 10.1007/s11886-012-0328-0

**Published:** 2013-01-11

**Authors:** Amedeo Chiribiri, Rene M. Botnar, Eike Nagel

**Affiliations:** Division of Imaging Sciences and Biomedical Engineering, King’s College London BHF Centre of Excellence, NIHR Biomedical Research Centre and Wellcome Trust and EPSRC Medical Engineering Centre at Guy’s and St. Thomas’ NHS Foundation Trust, The Rayne Institute, 4th Floor Lambeth Wing, St. Thomas’ Hospital, London, SE1 7EH UK

**Keywords:** Coronary angiography, Coronary venous system, Gadolinium, Magnetic resonance imaging

## Abstract

Although cardiovascular magnetic resonance allows the non-invasive and radiation free visualization of both the coronary arteries and veins, coronary vessel wall imaging is still undergoing technical development to improve diagnostic quality. Assessment of the coronary vessels is a valuable addition to the analysis of cardiac function, cardiac anatomy, viability and perfusion which magnetic resonance imaging reliably allows. However, cardiac and respiratory motion and the small size of the coronary vessels present a challenge and require several technical solutions for image optimization. Furthermore, the acquisition protocols need to be adapted to the specific clinical question. This review provides an update on the current clinical applications of cardiovascular magnetic resonance coronary angiography, recent technical advances and describes the acquisition protocols in use.

## Introduction

Despite substantial improvements in prevention and treatment [1], coronary artery disease (CAD), myocardial infarction and heart failure constitute the leading cause of death in the western world [2]. The current gold standard for the diagnosis of CAD is invasive coronary angiography, but the increasing prevalence of CAD and the relatively reduced diagnostic yield of invasive assessment [3] clearly indicate the need for noninvasive tests that could directly assess the integrity of the coronary lumen [4].

Cardiovascular magnetic resonance (CMR) theoretically provides a combined approach allowing the assessment of coronary arteries, cardiac function, viability, perfusion and cardiac anatomy. Moreover, magnetic resonance angiography (MRA) can potentially be used to directly visualize the coronary vessel wall [5], providing valuable integrated information for patients with coronary artery disease. Additionally, coronary MRA techniques allow the visualization of the anatomy of the coronary veins (CV), providing information for the optimal placement of pacemaker leads in cardiac resynchronization therapy in patients with heart failure [6, 7]. This review provides an update on current technical developments and clinical utilization of coronary MRA.

## Indications for Coronary MRA

Clinically accepted indications of coronary MRA are currently limited to the assessment of anomalies of the coronary arteries (class I indication) and aorto-coronary bypass grafts (class II indication). The use of MRA for the diagnosis of CAD on native coronary arteries has not yet entered clinical routine [8, 9].

### Coronary Anomalies and Aneurysms (Class I Indication)

Coronary MRA can accurately visualize the origin and the path of anomalous coronary vessels, as well as the presence and location of coronary aneurysms. Aneurysms are found, for example, in Kawasaki disease. The usually larger caliber of the vessels and the location of the aneurysms in proximal or ectatic segments facilitate their visualization. An important added benefit of coronary MRA is the absence of ionizing radiation, particularly important in younger patients, children and young women [8, 10].

### Coronary Bypass-Grafts (Class II Indication)

Bypass grafts can be visualized by coronary MRA with good image quality, benefiting from their stationary position, straight and known course, and large diameter compared to the coronary arteries. Several different approaches for the visualization of coronary bypass grafts were published in the literature, including spin echo [11–14] and gradient echo techniques. Moreover, the use of contrast agents for the enhancement of the blood signal [15, 16] allowed sensitivities for the detection of graft stenoses between 95 % and 100 %.

However, the presence of metallic clips along the course of the graft, causing signal voids due to susceptibility artifacts, is a common limitation of coronary bypass MRA. According to current guidelines, coronary MRA may be used at specialized centers to identify stenoses in coronary arterial bypass grafts [8].

### Coronary Artery Angiography for the Detection of CAD

Coronary MRA can visualize the proximal segments of the coronaries in nearly 100 % of cases. The best results are obtained with the left anterior descending (LAD) and the right coronary artery (RCA), while the left circumflex (LCX), which runs in the direct vicinity of the myocardium and at a larger distance from the coil elements, is frequently visualized with lower image quality and for a shorter course.

Previous studies reported an average visible length of 50 mm for the LAD, 80 mm for the RCA and 40 mm for the LCX [17–23]. There was an excellent agreement between the diameters of the proximal vessels measured by MRA and by invasive angiography [24].

The spatial resolution of coronary MRA is still lower than that of invasive coronary angiography, which limits the visualization of small branches and affects diagnostic accuracy concerning stenosis detection. This limitation explains the low specificity demonstrated in a recent international multicenter study [4], whereby coronary MRA was shown to have a high sensitivity (92 %) and a low specificity (59 %) for the detection of CAD. The diagnostic performance was much improved in a subanalysis of left main or three vessel disease (sensitivity 100 %; negative predictive value 100 %). A series of smaller single-center studies support these findings [17, 25–33].

A recent meta-analysis compared coronary MRA and multi-slice computed tomography (CT) for ruling out significant CAD in adults [34•]. CT was more accurate than MRA and therefore the authors concluded that CT, in its role of screening for CAD, can be considered as the preferred non-invasive alternative method to coronary catheterization. However, the advantage of coronary MRA is that it could be part of an integrated clinical protocol (including function, structure, perfusion and viability scans), allowing a more accurate evaluation of patients with known or suspected CAD.

Moreover, a more recent multicenter study from Japan showed that non-contrast-enhanced whole-heart coronary MRA at 1.5 T can detect significant CAD with high sensitivity (88 %) and moderate specificity (72 %). In particular, a negative predictive value (NPV) of 88 % indicates that whole heart coronary MRA can effectively be used to rule out CAD [35••]. Of note, the NPV reported by this multicenter trial is identical to the NPV of the CORE-64 CTA multicenter study [36], demonstrating the value of coronary MRA in ruling out coronary artery disease in patients with a pre-test probability of <20 % [37].

In a direct comparison between coronary MRA and CTA no significant difference was shown for the detection of coronary artery stenosis between 3 T MR and 64-slice CTA, although CTA showed a favorable trend toward higher diagnostic performance [38••].

### Coronary Vein Imaging

With the advent of resynchronization therapy, the assessment of the anatomy of the coronary venous system has become increasingly important, particularly for the pre-interventional identification of optimal placement site for the left ventricular lead of resynchronization devices. The same techniques used for coronary (artery) MRA can be used to visualize the coronary veins. Three-dimensional MR coronary vein angiograms can be overlaid onto real-time time acquired x-ray images, to improve guidance for catheter implantation [39, 40].

The integration of coronary venous anatomy and myocardial scar information may also guide left ventricular lead implantation remote from areas of scanned myocardium. Contrast agent enhanced MR can be used for the assessment of the course of the coronary sinus, the great cardiac vein, and their tributaries [6, 7, 41].

## Coronary Vessel Wall Imaging

The first magnetic resonance images of the coronary vessel wall were obtained by 2D fat saturated fast spin echo techniques [42, 43]. A double inversion recovery preparation is applied to obtain black-blood images improving the contrast between blood and vessel wall [44].

Recently, the double inversion recovery prepulse has been combined with fast gradient echo readout techniques [45], with spiral [46] and with radial acquisition trajectories [47].

Clinical studies demonstrated the ability of vessel wall imaging to detect outward positive remodeling with relative lumen preservation in patients with CAD and increased vessel wall thickness in patients with type I diabetes and renal dysfunction [48, 49]. As shown by Jansen and colleagues, non-contrast enhanced T1-weighed MR allows direct thrombus visualization in patients with acute myocardial infarction [50].

It is of particular interest that there are approaches that may allow visualization of inflamed plaques by means of delayed gadolinium enhancement techniques. Clinically approved contrast agents showed non-specific uptake in plaques both in patients with chronic angina [51] and in patients with acute coronary syndromes [52] and also in patients with systemic lupus erythematosus as sign of coronary inflammation or vessel wall activity [53]. Contrast uptake in patients with stable angina was associated with calcified or mixed plaques on MSCT while contrast uptake in patients with ACS the contrast uptake was transient and so most likely related to inflammation.

Several novel target specific contrast agents have been developed and tested in animal models. The accumulation of albumin binding blood-pool CA is associated with increased endothelial permeability and/or increased neovascularization [19, 54]. Furthermore, increased accumulation of iron-oxide particles (USPIO) also indicates increased endothelial permeability and vessel wall inflammation due to the presence of intraplaque macrophages [55, 56].

Such molecules and cells are providing targets for recently developed novel molecular contrast agents. These CA allow the selective visualization of inflammatory markers such as intercellular adhesion molecule-1 (ICAM-1), vascular adhesion molecule-1 (VCAM-1) or matrix metalloproteinase (MMP) [57, 58]. Additionally, the specific labeling of thrombi by a fibrin-specific contrast agent [59, 60] and the detection of extracellular matrix remodeling by targeting elastin has become an area of interest [61, 62]. Thus, molecular contrast agents may provide new opportunities for the identification of early atherosclerotic lesions as well as for the assessment of plaque vulnerability.

## Coronary MRA: Technical Considerations

The small caliber of the coronary vessels, as well as the elevated anatomical variability, cardiac and respiratory motion pose major challenges to coronary MRA and require dedicated techniques for image quality optimization.

### Ensuring Sufficient Image Contrast: Sequences, Spin Preparation and Contrast Agents

#### Sequences

The first approaches to coronary artery angiography were attempted by Edelman [63] and Manning [20] by 2-dimensional (2D) gradient-echo techniques. One slice was acquired in 16 heartbeats during a single breath-hold. Patients could breathe between acquisitions. 3D techniques adopting a whole heart or target volume approach became feasible after the introduction of navigator techniques. 3D sequences allow an increase of signal-to-noise ratio (SNR), enabling higher spatial resolutions. The main disadvantage is the reduced contrast between blood and the myocardium due to the reduction of in-flow effects. Hence there is a need for contrast enhancing spin preparations techniques [17, 64] to be used in combination with 3D gradient echo (GRE) or steady-state-free-precession (SSFP) sequences [65]. At 1.5 Tesla, the latter are preferred to T1 –weighted gradient echo sequences due to the higher SNR and improved contrast between blood and myocardium [65–67]. The use of SSFP sequences at 3 T is significantly limited by the prolongation of repetition times due to SAR limitations, the increased sensitivity to off-resonance effects and the need for higher flip angles. Gradient echo techniques appear as promising alternative [68].

#### Contrast-Enhancing Spin Preparations

For non-contrast enhanced imaging, spin preparation usually include fat suppression and T2-preparation. Fat saturation reduces the signal generated by the epicardial fat tissue, allowing a better delineation of the lumen of the coronaries [63, 64, 69]. In order to improve the contrast between the coronary lumen and the underlying myocardium, T2-preparation techniques can be used [17, 69] to reduce the myocardial signal, as blood and myocardium have similar T1 but different T2.

T2-preparation also suppresses deoxygenated venous blood due to the shorter T2 of deoxygenated hemoglobin. Contrast enhanced techniques are therefore preferred for coronary vein imaging.

Other solutions, such as spin-locking [70] and magnetization transfer techniques (MTC) [64], have also been proposed to improve the contrast between the vessel lumen and the myocardium. In particular, MTC does not affect the signal from venous blood and can therefore be used to visualize the coronary venous system without administration of CA [71]. The application of CA can further improve the contrast between blood and the surrounding tissues.

#### Contrast Agents

Different types of CA have been tested for coronary MRA, ranging from extracellular CA [72] to blood-pool CA [73–76], and CA with weak albumin binding [77, 78]. The decision about the CA to use depends on the availability of different CA, as well as on the balance between lumen-enhancing properties and the ability to provide information about myocardial scar as part of a combined ischemia/CAD diagnostic imaging protocol. Extracellular CA generates limited contrast between the vessel lumen and the myocardium due to the rapid extravasation in the interstitial space. Blood-pool contrast agents offer the highest contrast between the vessel lumen and the surrounding tissues but may face limitations for late enhancement imaging [9].

Spin preparation for CA enhanced MRA usually includes a saturation [79] or inversion prepulse [80] instead of the T2 preparation. The difference in T1 recovery between blood and myocardium after CA administration allows the generation of high contrast images.

### Improving Image Quality: Compensation of Cardiac and Respiratory Motion

#### Compensation of Cardiac Motion: ECG Triggering

To freeze cardiac motion, the acquisition of the *k*-space has to be synchronized with the cardiac cycle and needs to be limited to periods of minimal cardiac movement [81] occurring in end-systole (approximately 280–350 ms after the R wave) and in mid-diastole (immediately prior to atrial systole).

The choice of the trigger delay and the duration of the acquisition window depend on the patient’s heart rate, on the sequence used and on the structure to visualize (arteries or veins). A free breathing high temporal resolution cine scan in the 4-chamber view can be used to determine the resting period [81]. This is generally longer for the left compared to the right coronary system. For this reason, the resting period of the RCA should be selected for the acquisition of whole-heart scans.

#### Compensation of Respiratory Motion: Navigator

Since three-dimensional (3D) acquisitions take a long period of time to complete, they require the synchronization of image acquisition with the respiratory cycle. Respiratory motion artifacts can be minimized by prospective real-time navigator gating and correction techniques [82, 83], in which a pencil beam one-dimensional (1D) navigator is used to monitor the craniocaudal motion of the right hemidiaphragm [84] immediately prior to coronary image acquisition. If the position of the diaphragm falls within a certain acceptance window (usually 3–5 mm wide), the acquired data acquired is used, otherwise is rejected and has to be re-measured in the subsequent cardiac cycle. An acceptance window of 5 mm usually allows an efficiency approaching 50 % [85–87].

**Fig. 1:**
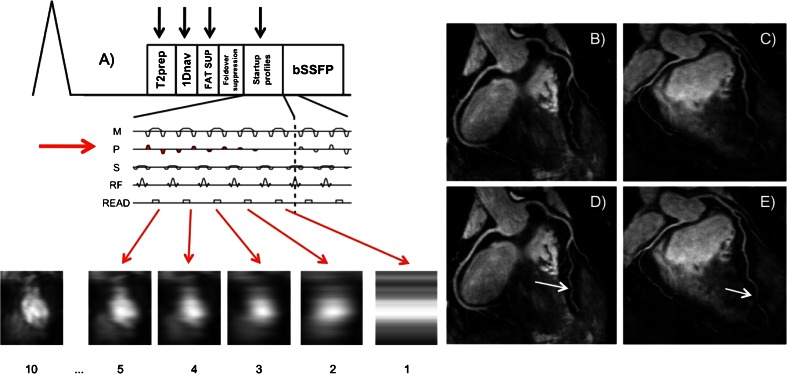
(**A**) Sequence diagram of the 2D navigator (2Dnav) used for prospective motion correction (Adapted from Henningsson et al. [88]). The 2Dnav is acquired from the startup profiles of the sequence and uses fat suppression (spectrally selective inversion recovery, FAT SUP) and fold-over suppression to suppress signal from epicardial fat and to reduce fold-over artifacts. Reconstruction and image registration are performed immediately after 2Dnav acquisition, which yields displacement information in FH and LR direction and is used for slice tracking. The 2Dnav can be combined with 2D or 3D gradient echo-based imaging sequences. The trailing 1D navigator is used for respiratory gating. The scanner gradients in measurement (M), phase encoding (P), and slice selection (S) are shown for the sequence, along with the radiofrequency pulses. (**B**,**C**,**D**,**E**) Examples of right and left coronary artery visualized with the 2Dnav sequence

However, respiratory motion remains the major impediment in a substantial amount of patients undergoing coronary MRA. Usually, 1D navigator techniques assume a constant linear relationship between diaphragmatic and cardiac motion. This assumption is not correct in a significant percentage of subjects, leading to suboptimal results. Recently, the use of 2D navigator has been proposed, allowing for the prospective correction of translational motion both in craniocaudal and left-right direction [88] (Fig. 1).

The use of image based navigators that directly track cardiac motion, navigators that monitor the movement of epicardial fat [89], scanning in prone position [90, 91] and the use of abdominal or thoracic banding [32, 91] have also been proposed.

Of note, a lower sensitivity to motion and better image quality can also be obtained by improving the speed of image acquisition because of a shorter acquisition window or shorter overall data acquisition time [9]. To this purpose, different approaches have been proposed, such as faster encoding of k-space by echo planar imaging (EPI) [92, 93], or more efficient k-space sampling using spiral [94] or less motion sensitive k-space sampling using radial trajectories [95]. However, none of these solutions have become an established technique for coronary MRA due to off-resonance sensitivity (EPI, spiral) or signal-to-noise penalty (radial).

Parallel imaging techniques such as SENSE [96] or SMASH [97] can reduce the overall MRA acquisition time while maintaining image quality.

## Coronary Vein Imaging: Technical Considerations

T2 preparation is not suitable for coronary vein imaging due to the shorter T2 values of deoxygenated venous blood. Current approaches to coronary vein MRA include non contrast-enhanced imaging by magnetization transfer (MTC) preparation [71, 98] or contrast enhanced MRA performed using blood-pool [6, 7, 41], extracellular [99], and CAs with weak albumin binding [100]. A slow infusion of a high relaxivity contrast agent during coronary vein MRA acquisition allows a good contrast between the vessel lumen and the surrounding tissues, with the possibility to acquire late gadolinium enhancement images after the redistribution of the contrast agent [100].

The optimal acquisition window for coronary vein imaging, as demonstrated by Nezafat and co-authors, is in end-systole, when the coronary vein diameter is maximal [71]. However, tachycardia and orthopnea cause difficulties with the ECG triggering and the asynchronous contraction of the LV makes the resting period different in independent segments of the chamber. In these patients, the acquisition parameters should therefore be adapted and data acquisition in end-diastole might be an alternative.

## Coronary Vessel Wall Imaging: Technical Considerations

Utilizing black-blood techniques allows the latest clinical MR scanners to provide a detailed visualization of the coronary artery wall, either in cross-section or along the path of the vessel. Partial volume effects are minimized in cross-sectional view, providing images suitable for accurate quantification of the vessel wall thickness. A long-axis view of the vessel wall provides instead a more extensive visualization and typically allows assessment of the proximal 5 cm [9].

CA can be used for selective plaque visualization and delayed enhancement images can show focal or diffuse uptake of contrast agent indicating either a fibrous plaque or inflammation.

The current application of coronary vessel wall imaging is restricted to research purposes but with developments such as plaque-targeting agents used for clinical purposes it may become part of routine CAD risk assessment and monitoring of treatment response, especially if plaque-targeting agents become available for clinical use.

## Conclusions

Cardiovascular magnetic resonance allows non-invasive and radiation free visualization of both the coronary arteries and veins, with the possibility of coronary vessel wall imaging. A major comparative advantage of MR is the possibility of a combined scanning protocol, investigating the anatomy of the coronaries as well as cardiac function, viability, stress perfusion and cardiac anatomy in the same study, providing valuable integrated information for patients with coronary artery disease and heart failure.

Coronary MRA may be indicated for the visualization of anomalies of the origin and course of the coronaries (class I indication) as well as to visualize coronary bypass grafts (class II indication) and may potentially be used to exclude CAD in selected populations of patients.

Ongoing technical developments continue to improve the robustness of the techniques.

## List of Abbreviations

2D: 2-dimensional
3D: 3-dimensional
CA: contrast agent
CAD: coronary artery disease
CMR: cardiovascular magnetic resonance
CT: computed tomography
CV: coronary veins
EPI: echo planar imaging
ICAM-1: intercellular adhesion molecule - 1
LAD: left anterior descending (coronary artery)
LCX: left circumflex (coronary artery)
MMP: matrix metalloproteinase
MRA: magnetic resonance angiography
MTC: magnetization transfer contrast
RCA: right coronary artery
SAR: specific absorption rate
SENSE: sensitivity encoding
SMASH: simultaneous acquisition of spatial harmonics
SNR: signal-to-noise ratio
SSFP: steady state free precession
USPIO: ultra-small super paramagnetic iron oxide
VCAM-1: vascular adhesion molecule – 1
